# Optimal nerve release range and transposition distance in anterior ulnar nerve transposition for cubital tunnel syndrome: an anatomical study

**DOI:** 10.1016/j.jseint.2025.07.003

**Published:** 2025-08-05

**Authors:** Kohei Hirukawa, Koji Sukegawa, Yukie Metoki, Takuya Tada, Tomomi Mizuhashi, Kentaro Uchida, Kenji Onuma, Yuya Otake, Motoyuki Ogawa, Gen Inoue, Masashi Takaso

**Affiliations:** a Department of Orthopedic Surgery, Kitasato University School of Medicine, Sagamihara City, Japan; b Department of Graduate School of Medical Sciences, Kitasato University, Sagamihara City, Japan; c Department of Clinical Anatomy, Research and Development Center for Medical Education, Kitasato University School of Medicine, Sagamihara City, Japan; d Department of Anatomy, Kitasato University School of Medicine, Sagamihara City, Japan

**Keywords:** Anterior transposition, Cubital tunnel, Elbow joint, Ulnar nerve

## Abstract

**Background:**

Cubital tunnel syndrome, the second most common entrapment neuropathy, results from ulnar nerve compression at the medial epicondyle (ME). Conservative treatment often fails, requiring surgery. Transposition is an effective but invasive procedure, necessitating precise anatomical guidelines. We aimed to investigate the optimal anatomical measurements for ulnar nerve transposition to facilitate standardization of the technique.

**Methods:**

We examined 12 upper limbs from 6 fresh-frozen cadavers with elbows flexed at 60° and forearms supinated. The optimal transposition distance was defined as the distance from the ME to where the ulnar and median nerves run parallel. We measured this distance; pivot points A and B (where the ulnar nerve's course changes after transposition); crossing points over the medial intermuscular septum and flexors (A′, B′); and ulnar nerve branch positions from the inferior border of the ME.

**Results:**

After excluding 3 outliers using the interquartile range method, 10 limbs were analyzed. The transposition distance was 16.6 ± 3.3 mm. Pivot points A and B were 53.8 ± 6.5 mm and 50.2 ± 9.1 mm from the ME. Crossing points A′ and B′ were 39.6 ± 7.9 mm and 40.2 ± 6.7 mm. Nerve branches were 21.1 ± 6.0 mm, 32.4 ± 14.8 mm, and 50.9 ± 24.6 mm from the inferior border of the ME.

**Conclusion:**

Anterior transposition shifts the ulnar nerve 17 mm anterior to the ME. To prevent kinking, dissection should extend 54 mm proximally and 50 mm distally, totaling approximately 105 mm. These measurements can guide intraoperative planning and emphasize the need for direct visualization to ensure safe and effective anterior transposition.

Cubital tunnel syndrome (CuTS) is the second most common compressive neuropathy after carpal tunnel syndrome. This syndrome is often accompanied by ischemia, and it is caused by various degrees of compression, friction, and elongation of the ulnar nerve at the medial aspect of the elbow.^5^^,^^9^^,^^10^ Clinically, CuTS presents with sensory disturbances—such as numbness and pain on the ulnar side of the little and ring fingers—and decreased grip strength due to intrinsic muscle atrophy. These symptoms typically respond poorly to conservative treatment and tend to progress.^9^^,^^10^

Various anatomical structures around the medial epicondyle (ME) have been associated with CuTS, including Osborne's band, Osborne's ligament, the cubital tunnel retinaculum, Osborne's fascia, the arcuate ligament of Osborne, the ligamentum epitrochleoanconeus, the common flexor aponeurosis, and the deep flexor–pronator aponeurosis.^4^^,^^7^^,^^8^^,^^13^^,^^16^^,^^20^^,^^21^^,^^26^^,^^27^ However, difficulties in distinguishing these structures anatomically, as well as differences in interpretation among surgeons, have led to confusion.^27^ Proximal to the ME, the medial intermuscular septum (MIS), the medial head of the triceps brachii, and arcade of Struthers have also been implicated in the development of CuTS.^6^^,^^25^ Furthermore, factors such as excessive use during sports or occupational activities, progression of osteoarthritis due to aging, residual elbow deformities following pediatric fractures (such as valgus or varus malunions), and subluxation of the ulnar nerve with snapping triceps predispose patients to ulnar neuropathy.^1^^,^^8^^,^^14^^,^^22^^,^^23^ Systemic diseases such as diabetes and hypothyroidism may further increase nerve vulnerability and contribute to the development of CuTS.

Conservative management of CuTS includes lifestyle modifications and splint therapy; however, surgical intervention is considered when these measures fail or symptoms progress.^11^ Surgical options include medial epicondylectomy (King procedure), neurolysis, simple decompression, and subcutaneous or submuscular anterior transposition of the ulnar nerve.^3^^,^^12^ According to Mowlavi et al, treatment selection depends on the severity of CuTS; in mild cases, all treatment modalities yield comparable satisfaction. Conversely, in moderate to severe cases, anterior transposition produces superior outcomes compared with simple decompression, suggesting that anterior transposition is the preferred option.^19^

Despite its efficacy, anterior transposition is more invasive than other techniques. To avoid unnecessary invasiveness, clear anatomical criteria for the optimal extent of ulnar nerve release and the degree of transposition are required. Incomplete release, abnormal routing, kinking, and perineural scarring are known causes of recurrence or persistent symptoms, indicating that the choice of surgical technique and the accuracy of intraoperative maneuvers are critical.^15^ Therefore, establishing the appropriate release range and degree of transposition to maintain the nerve's natural course is essential.

In this study, we aimed to anatomically elucidate the optimal extent of nerve release and degree of transposition in anterior transposition of the ulnar nerve for CuTS, thereby contributing to the standardization of the surgical technique and aiding procedure selection.

## Materials and methods

This study was conducted after obtaining approval from our institutional ethics committee (approval number B18-276).

Twelve upper limbs from 6 fresh-frozen cadavers (3 male and 3 female) were used. The mean age at death was 84 years (range, 63-97 years). Cadavers with gross deformities around the elbow or a restricted range of motion were excluded. Gross deformity was defined as a visually apparent abnormal joint contour or alignment of the humerus and ulna, such as post-traumatic malunion or advanced osteoarthritic change. None of the specimens were excluded based on these criteria.

Humeral length and elbow range of motion were recorded before dissection. All specimens were amputated at the shoulder joint. Measurements were taken with the elbow flexed to 60°, a position commonly adopted during anterior transposition procedures in clinical settings. This angle was also selected based on a previous study by Abrams et al, which reported that deep flexion might induce bowstringing of the ulnar nerve in cadaveric models, impairing measurement accuracy.^2^ A skin incision was made centered on the ME, and the soft tissues were carefully dissected to expose the medial aspect of the elbow.

The ulnar nerve was identified and marked proximal to the ME. Proximally, the arcade of Struthers was released, while distally, Osborne's ligament, Osborne's fascia, and the ligamentum epitrochleoanconeus were released to perform neurolysis of the ulnar nerve.^16^^,^^21^^,^^25^^,^^26^ Dissection was extended distally to preserve the ulnar nerve branches until the level of the ulnar wrist flexors was reached. The median nerve, located centrally on the anterior aspect of the elbow, was also identified and exposed.

**Figure 1:**
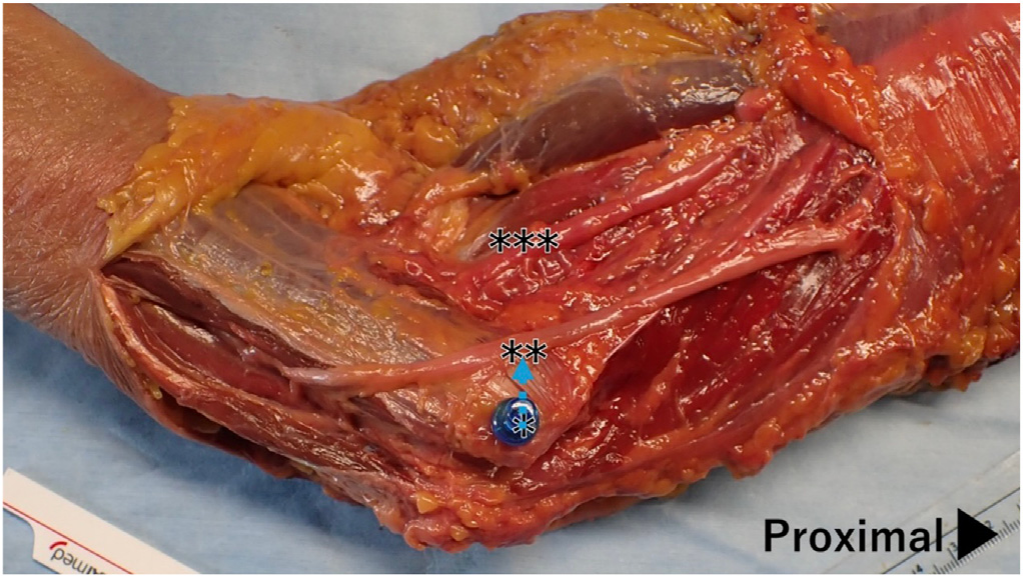
Implementation of ulnar nerve anterior transposition.Neurolysis of the ulnar nerve is performed while preserving the nerve branches. The median nerve, located in the central ventral aspect of the elbow, is identified and exposed. Based on the work of Leamonth^17^ and Dellon,^8^ the optimal distance for the ulnar nerve transposition (blue arrow) is measured. ∗ Medial epicondyle of the humerus; ∗∗ ulnar nerve; ∗∗∗ median nerve.

Based on the reports by Dellon^8^ and Leamonth,^17^ the optimal transposition was defined as the movement required for the ulnar nerve to run parallel to the median nerve; the transposition distance was measured as the distance from the ME to the point of parallel alignment (Fig. 1). If any branches of the ulnar nerve hindered transposition, the minimal necessary neurolysis was performed; when required, these branches were also transected.^8^^,^^17^

**Figure 2:**
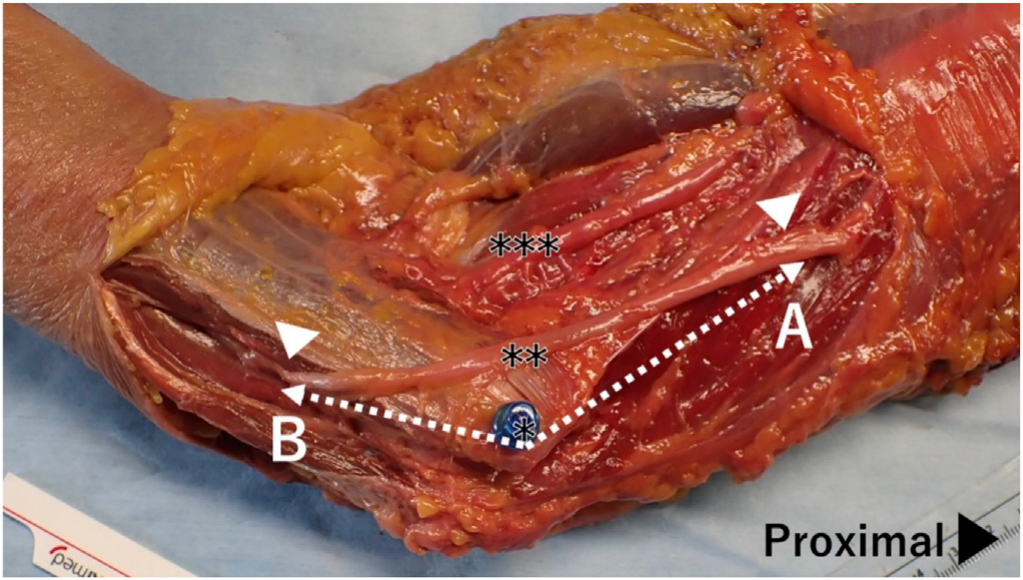
Pivot points of the ulnar nerve. From the ME of the humerus, the points where the original course of the ulnar nerve changes due to anterior transposition are measured as the proximal (A) and distal (B) pivot points. ∗ ME of the humerus; ∗∗ ulnar nerve; ∗∗∗ median nerve. *ME*, medial epicondyle.

**Figure 3:**
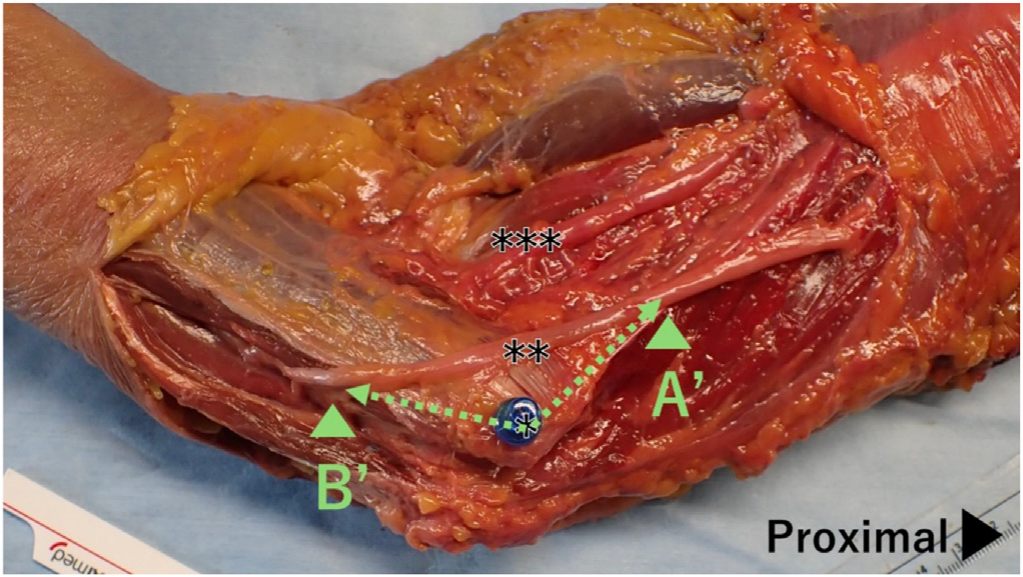
Ascension points of the ulnar nerve. For the proximal ascension, the distance from the ME to the point where the ulnar nerve ascends onto the humerus (A′) is measured. For the distal ascension, the distance from the ME to the point where the ulnar nerve ascends onto the flexor group (B′) is measured. ∗ ME of the humerus; ∗∗ ulnar nerve; ∗∗∗ median nerve. *ME*, medial epicondyle.

**Figure 4:**
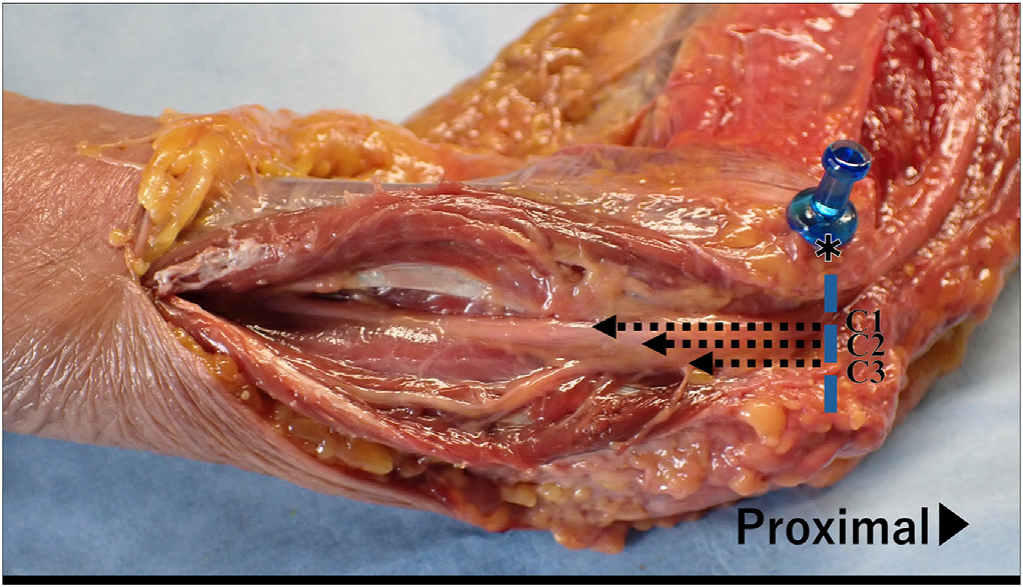
Positions of the ulnar nerve branches. The distances from the inferior border of the medial epicondyle to each ulnar nerve branch in the distal direction are measured and labeled, from proximal to distal, as C1, C2, and C3. ∗ Medial epicondyle of the humerus.

After anterior transposition of the ulnar nerve, the following measurements were obtained: from the ME, the proximal and distal points at which the nerve's original course changed after transposition were recorded as the proximal pivot point (A) and distal pivot point (B), respectively (Fig. 2). The positions at which the transposed ulnar nerve ascended onto the MIS (point A′) and onto the flexor muscle group (point B′) were also measured (Fig. 3). Finally, the distances from the inferior border of the ME to each distal branch of the ulnar nerve were measured sequentially as C1, C2, and C3 (Fig. 4).

After completing the measurements, elbow flexion and extension were performed to visually confirm that the ulnar nerve was not constricted and its course remained unobstructed. The nerve was transposed into the submuscular layer of the flexor–pronator group, and the elbow was flexed and extended to confirm that no buckling, kinking, or protrusion occurred in any case. This assessment was not based on a quantitative method; however, in the context of practical intraoperative assessment, it is a meaningful dynamic evaluation.

All measurements were consistently performed by a single board-certified orthopedic surgeon specializing in hand surgery to minimize measurement error. A digital caliper manufactured by Mitutoyo (model CD-P20S; Sakado, Japan) [minimum resolution, 0.01 mm; maximum permissible error, ±0.02-0.04 mm] was used for all measurements.

For statistical analysis, the correlation between humeral length and each measured parameter (transposition distance, ME-A, ME-B, ME-A′, and ME-B′) was assessed. Outliers were identified using the interquartile range (IQR) method. Specimens with values exceeding 1.5 × IQR in two or more independent variables were classified as outliers and excluded from further analysis to enhance statistical robustness. Next, the Shapiro–Wilk test was used to determine whether each measurement followed a normal distribution (*P* > .05, indicating a normal distribution; *P* ≤ .05, indicating a non-normal distribution). Finally, assuming a normal distribution, Pearson correlation coefficient was calculated to evaluate the relationship between humeral length and each measured parameter.

## Results

Using the IQR method, 3 specimens that exhibited outlier values in two or more measurement parameters were excluded from the analysis to minimize the influence of potential measurement error or anatomical variability (see Table I).

Excluded specimen 1 (female, 84 years old) showed extremely high values in ME-A (84.63 mm) and ME-A′ (69.71 mm). Excluded specimen 2 (female, 97 years old) showed extremely low values in ME-A′ (25.19 mm) and ME-B′ (22.19 mm). Excluded specimen 3 (male, 81 years old) showed extremely high values in ME-A (77.07 mm) and ME-B (72.46 mm). These deviations may have been influenced by individual anatomical variations in the course of the median and ulnar nerves and differences in intermuscular structures.

As a result, the final analysis included 9 upper limbs from 6 cadavers.

The average elbow range of motion was 138° ± 9° for flexion (range, 120°-150°) and −6° ± 6° for extension (range, −15° to 0°). The humeral length averaged 273 ± 19 mm (range, 249-315 mm). Subluxation of the ulnar nerve during elbow flexion was observed in only 1 limb. The distance for ulnar nerve transposition was 16.6 ± 3.3 mm (range, 11.8-23.2 mm).

After anterior transposition, the proximal pivot point A and the distal pivot point B were located 53.8 ± 6.5 mm (range, 42.5-63.6 mm) and 50.2 ± 9.1 mm (range, 38.7-65.7 mm) from the ME, respectively. The distance from the ME to the point where the transposed nerve ascended onto the MIS (A′) was 39.6 ± 7.9 mm (range, 25.4-54.5 mm), and the distance to the point where it ascended onto the flexor muscle group (B′) was 40.2 ± 6.7 mm (range, 27.6-53.2 mm).

The positions of the ulnar nerve branches were: C1, 21.1 ± 6.0 mm (range, 11.9-31.4 mm); C2, 32.4 ± 14.7 mm (range, 17.3-69.8 mm); and C3, 50.9 ± 24.6 mm (range, 34.9-105.0 mm).

The Shapiro–Wilk test confirmed that all measured parameters were normally distributed (*P* > .05). Pearson correlation coefficient revealed a weak positive correlation between humeral length and the M-B distance (r = 0.205). In contrast, correlations for transposition distance, ME-A, and M-–B′ were very weak (r = 0.055-0.180). The ME-A′ distance showed a weak negative correlation with humeral length (r = −0.299).

## Discussion

In this study, we aimed to standardize the surgical technique of anterior transposition of the ulnar nerve by examining the transposition distance, extent of nerve release, and branching positions. The procedure was performed according to the submuscular transposition technique described by Dellon and Leamonth.^8^^,^^17^ With the elbow flexed at 60° and the forearm supinated, the ulnar nerve was transposed anteriorly until it ran parallel to the median nerve.

Our results suggested that the ulnar nerve should be transposed approximately 17 mm anterior to the ME, with approximately 54 mm of proximal release and 50 mm of distal release, totaling approximately 105 mm. In addition, the nerve crossed the MIS and flexor muscle group at approximately 40 mm from the ME. These values may serve as useful guidelines for determining the length of the skin incision and minimizing the risk of iatrogenic injury to the nerve branches.

Smetana et al used 11 limbs from fresh-frozen cadavers and measured ulnar nerve tension at various wrist and elbow flexion angles, reporting that nerve tension was minimized at 60° elbow flexion.^24^ This suggests that the 60° flexion position is optimal for safe nerve transposition with minimal tension. Excessive tension can impair blood flow and cause tissue damage, potentially hindering postoperative recovery.

Abrams et al^2^ also reported that in deep elbow flexion, the ulnar nerve becomes taut in the subcutaneous plane and assumes a straighter course, a phenomenon termed bowstringing. This may result in a shortened linear distance between the pivot points, potentially leading to an underestimation of the required transposition and release distance. Measurements in the present study were taken with the elbow at 60° flexion to avoid this risk and ensure reproducibility.

The median nerve arises from the lateral and medial cords of the brachial plexus, travels centrally along the anterior aspect of the elbow, and enters the carpal tunnel. In contrast, the ulnar nerve, arising from the medial cord, passes posterior to the ME at the elbow and travels ventrally in the forearm to enter Guyon canal at the wrist. Thus, aligning the ulnar nerve parallel to the median nerve provides a more anatomically efficient course. In addition, although anatomical differences exist among individuals, our findings were generally consistent. Thus, using the median nerve as a reference point enables adaptable yet standardized transposition planning.

Li et al examined 18 upper limbs from fresh-frozen cadavers to describe the distribution, length, and origins of the ulnar nerve's vascular supply, including the superior ulnar collateral, inferior ulnar collateral, and posterior ulnar recurrent arteries.^18^ They determined that the maximal tension-free transposition distance preserving these vessels was 18 ± 6 mm. Our findings are consistent with this range, indicating that a transposition distance of approximately 10-20 mm is sufficient to preserve vascular supply while allowing adequate nerve mobilization.

According to Kholinne et al, recurrence or persistent symptoms in CuTS may be caused by perineural scarring, incomplete decompression, or instability/kinking of the ulnar nerve.^15^ Felder et al highlighted several critical distal elbow structures relevant to anterior transposition, including branches of the medial antebrachial cutaneous nerve, Osborne's fascia, branches to the flexor carpi ulnaris (FCU), vascular branches from the ulnar artery, the distal MIS between FCU and flexor digitorum superficialis, and the conjoined origins of the flexor–pronator group with the investing fascia of the flexor digitorum superficialis.^11^^,^^27^ Proper management of these structures is essential to minimize complications.

In our study, anterior transposition of approximately 17 mm placed the proximal and distal pivot points at approximately 54 mm and 50 mm from the ME, respectively. This indicates that about 105 mm of nerve release is needed for a tension-free and smooth course. Furthermore, the nerve was found to ascend the MIS and flexor muscle group at approximately 40 mm from the ME, suggesting that proximal resection of the MIS and partial distal release of the FCU fascia are necessary. In submuscular transposition, dissection must extend beyond these points to allow adequate release and reflection of the flexor–pronator group. These anatomical insights provide practical guidelines for achieving vascular preservation and a tension-free nerve course.

To enhance statistical validity, 3 specimens showing significant outlier values across multiple parameters were excluded based on the IQR method. These deviations may have resulted from measurement error, anatomical variation, or environmental conditions. Nevertheless, the overall trends remained consistent after excluding the outliers.

All parameters followed a normal distribution based on the Shapiro–Wilk test, justifying the use of parametric tests. Pearson correlation analysis demonstrated only weak correlations between humeral length and the measured values, suggesting limited clinical relevance.

Future directions include assessing the impact of varying elbow flexion angles on nerve transposition distance and extent of release. In clinical practice, ulnar nerve transposition is often performed at approximately 60° of elbow flexion; however, the influence of deeper flexion on surgical planning warrants further study. In addition, future studies should address anatomical variability, long-term postoperative nerve function, and the use of preoperative imaging to delineate nerve branching and crossover points. These efforts will contribute to the development of safer and more effective surgical procedures for CuTS.

### Limitations

This study had some limitations. First, the sample size was limited to 12 upper limbs from fresh-frozen cadavers, which may restrict the generalizability of the results.

Second, as an anatomical study using cadaveric specimens, physiological conditions such as muscle tone and blood flow could not be fully replicated. In particular, vascular supply to the ulnar nerve was not directly assessed; rather, vascular preservation was inferred indirectly from anatomical observations. Third, all measurements were performed with the elbow fixed at 60° flexion and the forearm supinated, which does not represent the full range of elbow motion encountered in daily activities. Dynamic behavior and release requirements of the nerve in other positions or during motion were not evaluated. Future studies should investigate measurements at various elbow flexion angles and incorporate dynamic assessments to acquire more clinically relevant data.

Fourth, individual anatomical differences, such as age, sex, and ethnicity, were not thoroughly analyzed. These factors may influence the required transposition distance, extent of release, and branching positions and should be considered in future research.

Fifth, outliers were excluded based on the IQR method using a conservative criterion: specimens were excluded only if they showed outlier values in two or more measurement parameters. We excluded 3 specimens based on this rule. This approach improved statistical robustness by minimizing the influence of atypical values; however, it remains unclear whether the excluded data reflected measurement error or true anatomical variation. Therefore, biologically meaningful information may have been lost. Notably, the excluded specimens exhibited marked deviations in values such as ME-A and ME-B distances, suggesting the influence of structural anatomical variation.

Sixth, this study did not evaluate the potential impact of muscle mass (hypertrophy of the biceps or triceps brachii) or the carrying angle of the elbow on nerve mobility or transposition distance. These anatomical and biomechanical factors may influence the path and behavior of the ulnar nerve during elbow flexion and should be addressed in future investigations.

## Conclusion

In this study, we clarified the distance of ulnar nerve transposition and the required extent of release, providing quantitative data that may contribute to preoperative planning and standardization of the surgical technique for anterior transposition in CuTS. Detailed anatomical analysis using fresh-frozen cadavers allowed for clear identification of the ulnar nerve branches and the points where the nerve ascends onto the MIS and the flexor muscle group, which is valuable for enhancing the safety of the surgical approach.

These findings are consistent with previous anatomical studies and support the validity of the anterior transposition model employed. This study offers reference data for surgical design, enabling sufficient transposition with minimal release while preserving the natural course of the nerve. These measurements may serve as helpful intraoperative indicators for assessing nerve trajectory and tension.

However, the present study was based on a static anatomical model and did not assess nerve behavior under conditions of deep elbow flexion or dynamic positioning. Thus, the measurements provided should be interpreted as supportive indicators and not absolute values, and intraoperative judgment remains essential. The surgeon must confirm the path, tension, and gliding characteristics of the nerve through direct visualization when determining the appropriate extent of release. Therefore, while these values provide helpful anatomical references, they must not be used as rigid criteria. Over-reliance on them without confirming nerve tension, mobility, and surrounding structures could lead to insufficient decompression, residual kinking, or recurrent symptoms.

**Table I:** Boxplots showing 5 measurement parameters after excluding 3 outlier limbs based on the IQR method.

*IQR*, interquartile range.

Boxes indicate the IQR, the red line shows the median, and whiskers represent 1.5 × IQR. Circles denote remaining outliers.

## Declaration of generative AI and AI-assisted technologies in the writing process

ChatGPT (OpenAI, San Francisco, CA, USA) was used to assist with English editing and the creation of summary tables. The authors reviewed and revised all content as needed and take full responsibility for the content of the publication.

## Disclaimers

Funding: No external funding was received for this study.

Conflicts of interest: The authors declare no conflicts of interest related to this study. The views expressed in this article are those of the authors and do not necessarily reflect the official policies or positions of their affiliated institutions.
